# Emerging Roles of Branched-Chain Amino Acid Supplementation in Human Diseases

**DOI:** 10.1155/2014/235619

**Published:** 2014-11-12

**Authors:** Nahid Tamanna, Niaz Mahmood

**Affiliations:** ^1^Graduate Program in Biological Sciences, University of Manitoba, Winnipeg, MB, Canada R3T 2N2; ^2^Graduate Program in Biochemistry and Medical Genetics, University of Manitoba, Winnipeg, MB, Canada R3E 0J9

## Abstract

The branched-chain amino acids (BCAAs), namely, valine, leucine, and isoleucine, are indispensable amino acids required for body protein synthesis. Unlike other amino acids, the BCAAs are primarily catabolised in the extrahepatic tissues. The BCAAs play role in regulation of protein synthesis and turnover as well as maintenance of the body glutamate-glutamine level. In strenuous and traumatic conditions, the BCAAs are oxidized which limits their availability in tissues. Such condition affects the body glutamate-glutamine pool and protein synthesis mechanisms. Thus BCCA supplementation is emerging as a nutritional strategy for treating many diseases. Many studies have found that BCAA administration is able to improve the health status of the patients suffering from different diseases even though there are conditions where they do not exert any effect. There are also some reports where elevated BCAAs have been shown to be associated with the pathogenesis of diseases. In this review, we have discussed the implication of BCAA supplementation in different pathological conditions and their relevant outcomes.

## 1. Introduction

The branched-chain amino acids (BCAAs), valine, leucine, and isoleucine, are three of nine essential amino acids that are not synthesized by our body and therefore must be obtained from diet. Approximately 35% of indispensable muscle proteins and 40% of total amino acids required by mammals are comprised of these BCAAs [1]. The three BCAAs either together or leucine alone can stimulate protein synthesis [2] and can also inhibit protein degradation depending on the context [3].

Although most of the amino acids are degraded in the liver, BCCAs are primarily catabolised by extrahepatic tissues (muscle, adipose, kidney, and brain). Catabolism of these amino acids is initiated by transamination reaction with alpha-ketoglutarate to form glutamate and branched-chain keto acids (BCKAs). Then the glutamate is converted to glutamine by the action of glutamine synthetase enzyme. Glutamine has numerous functions including muscle protein synthesis [4], maintenance of acid-base balance in the kidney [5], glutathione (GSH) production [6, 7], expression of heat shock proteins (HSPs) [8], and removal of toxic ammonia from the tissues.

Thus, decline in the level of the BCCAs may affect the body glutamate-glutamine pool leaving the tissue more vulnerable to oxidative stress. Experimental evidences suggest that sepsis, cancer, trauma, and burns enhance oxidation of BCAAs and decline BCCAs level [9, 10]. In these circumstances, BCAA supplementation is necessary to maintain the physiologically relevant level of BCAAs in the body. Even though BCCA supplementation has been reported to be beneficial for the treatment of some of the diseases [11–13], there are also diseases where BCAAs should be restricted [13]. In this review we have summarized the implication of BCAAs in treating some diseases and also discussed the conditions when BCAAs should be limited.

## 2. BCAAs for Treating Liver Diseases

Several studies have demonstrated that administration of amino acid enriched with BCAAs can reduce protein loss, support protein synthesis, and improve the nutritional status of patients with hepatic illness. Some of them are summarized in the following texts.

### 2.1. BCAAs for Liver Cirrhosis

It has been reported that patients with liver cirrhosis suffer from protein malnutrition despite taking adequate food. This is manifested by skeletal muscle loss and hyperalbuminemia [14–16]. In a prospective study, to see the effects of BCAAs on decompensated cirrhosis, 646 patients were orally administered with either 12 g of BCAAs or diet therapy [13]. Compared with the diet group, oral administration of BCAAs significantly improved serum albumin concentration (*P* < 0.5) and health-related quality of life in patients with decompensated cirrhosis with an adequate daily food intake [13]. In another study, seven cirrhotic patients (age: 70 ± >6 years; M/F = 4/3; etiology: hepatitis C in six and non-B/non-C hepatitis virus in one; Child-Pugh classification: A in six and B in one) were given 4 g BCAA after each meal for 8 weeks [14]. This improved the oxidized/reduced state of serum albumin [14]. In a separate study, BCAAs were administered for 1 year to 174 patients with advanced cirrhosis. Consistent with the previous studies, the BCAAs were found to stabilize liver function test and overall health status [16]. Taken together, these studies suggest that BCAA supplementation has positive effect on patients with liver cirrhosis.

### 2.2. BCAAs for Hepatic Encephalopathy (HE)

Hepatic encephalopathy (HE) occurs when the liver is unable to remove toxins from the blood which ultimately results in the loss of brain function. In an effort to investigate the long-term effect of BCAA supplementation after an episode of HE, a randomized, double-blind, multicenter study was conducted among 116 patients with cirrhosis and a previous episode of hepatic encephalopathy [17]. All patients received a standard diet of 35 kcal/kg per day and 0.7 g of proteins/kg per day and a supplement of 30 g of BCAA (BCAA group) or maltodextrin (MDX group) for 56 weeks. Although the actuarial risk of remaining free of HE did not differ between groups (BCAA = 47%, MDX = 34%, *P* = 0.274), patients in the BCAA group exhibited a better outcome on two neuropsychological tests and also showed an increase in the mid-arm muscle circumference. BCAAs administration, however, could not prevent the recurrence of HE [17]. Gluud et al. (2013) also found that BCAAs administration can improve hepatic encephalopathy, but it does not have any effect on mortality [18].

### 2.3. BCAAs for Liver Cancer

BCAAs have also been supplemented for liver cancer. In a large study comprising 124 patients who were undergoing perioperative nutritional support after hepatectomy due to hepatocellular carcinoma, they were assessed for clinical outcome of BCAAs supplementation [19]. In addition to oral diet, sixty-four patients were randomly assigned to receive perioperative and postoperative intravenous nutritional support (35% BCAAs) for two weeks (7 days before surgery and 7 days after surgery), whereas the rest of the sixty patients were randomly assigned to a control group having a normal oral diet. A significant decrease of postoperative morbidity in the nutrition-supported group (34%) was observed compared with the control group (55%). There was also decreased use of diuretic therapy to control ascites in the perioperative-nutrition group (25%) compared to the control group (50%), and no weight loss was manifested in the perioperative-nutrition group (0 kg) compared with the control group (1.4 kg). However, hospital mortality rate did not differ between the perioperative-nutrition group (8%) compared with the control group (15%, *P* = 0.30). In the postoperative period, there was no significant difference between the two groups in terms of prothrombin time, serum bilirubin measurements, and liver-enzyme levels. However, the serum aspartate aminotransferase level was different between the two groups. Plasma glucose, serum urea, serum transferrin, serum prealbumin, and serum retinol-binding protein levels were significantly higher in the perioperative-nutrition group compared to the control group during most of the postoperative days [19].

In another study, 150 patients who had undergone possibly curative hepatic resection, two to three weeks after operation, were randomly assigned into control and supplemented groups [20]. Control group received their usual meal whereas supplemented group drank 50 g of Aminoleban EN (Otsuka Pharmaceutical Company, Tokyo, Japan) along with their usual diet for a year. Aminoleban is a good source of BCAAs. It also contains small amount of other amino acids, ten types of mineral and fourteen types of vitamin. BCCAs improved both body weight and tremor in the supplemented group. Higher Fischer's molar ratio was found in the supplemented group. Although long term BCCAs supplementation improves the health performance in the treated group, it was not able to decrease the mortality rate compared to the controls [20].

In a separate prospective study, patients having hepatocellular carcinoma were divided into two groups [21]. In addition to normal diet study group received Aminoleban EN for 12 weeks, whereas control group received an isonitrogenous and isocaloric diet. The study group had a shorter hospital stay and had a significantly higher level of haemoglobin, sodium, and albumin and lower level of bilirubin during the postoperative course. However, no significant difference was observed in terms of neuropsychiatric symptoms or Karnofsky performance score and also there was no difference found in gastrointestinal symptoms between two groups. There was not any noticeable adverse reaction upon the administration of Aminoleban, and the two groups also did not show any difference in terms of morbidity and mortality. From this study, Meng et al. (1999) concluded that Aminoleban EN is safe to administer and its administration in the early operative period contributes to a shorter hospital stay and quicker improvement of liver function in the early postoperative period [21].

### 2.4. Administration of BCAAs in Nonalcoholic Steatohepatitis

Nonalcoholic steatohepatitis (NASH) is often defined as “silent” liver disease which is characterized by fatty liver, inflammation, and degeneration of hepatic cell, with or without fibrosis and necrosis [22]. It resembles alcoholic liver disease but occurs in people who drink either little or no alcohol. Li et al. (2013) have studied the effects of BCAAs on lipid metabolism over an 8-week experimental period on laboratory rats [23]. They fed high fat diet to gonadectomised rats and assessed body composition, tissue histology, plasma lipid indices, and hormone levels. Although, in BCAAs treated rats, the body weight was not significantly decreased compared to the controls, supplementation of BCAAs was able to decrease mesenteric fat (*P* < 0.05) in treated rats. Plasma lipid levels and fat deposition were also decreased in the liver of treated rats. During the 4th week of experiment, the control rats displayed macrovesicular steatosis while BCAA-treated rats had only macrovesicular droplets in their hepatocytes. At the 8th week, when the untreated rats develop profound cirrhosis, BCAA-treated rat livers remained in the macrovesicular stage of steatosis. BCAA-treated rats had higher blood glucose and plasma insulin levels (*P* < 0.05). In the BCAA-treated group, liver blood flow was also improved by increasing mean arterial blood pressure and decreasing portal pressure, which helped delay the change in blood flow pattern to that of cirrhosis. This study demonstrated that BCAAs can lower fat deposition in rats fed a high-fat diet [23] which might be a potential strategy to treat NASH in humans.

## 3. Plausible Mechanism of Preventing Cancer by BCAAs

Mammalian target of rapamycin (mTOR) is identified as a promising therapeutic target for the treatment of a number of types of cancer [24, 25]. It was found that BCAAs, especially leucine, can regulate protein synthesis through mTOR activities [26]. Nakano et al. [12] found that human cancer cells HepG2 and U2OS cultured in BCAAs medium were capable of synthesizing high level of albumin and had greater capacity to induce premature senescence and mTORC1 activities. The protein levels of p21, a p53 target and well-known gene essential for the execution of cellular senescence, were upregulated in the presence of BCAAs. It is suggested that BCAAs possibly contribute to tumor suppression by enhancing cellular senescence mediated through the mTOR signalling pathway [12].

## 4. BCAAs for Skeletal Muscle

Even though leucine supplementation enhances protein synthesis and inhibits protein degradation, this response is not always consistent. The effects of branched-chain amino acids on whole-body and skeletal muscle amino acid kinetics were studied by intravenous administration of l-[*ring*-2, 6-^3^H] phenylalanine and l-[1-^14^C] leucine to 10 post absorptive human healthy subjects [27]. Supplementation of leucine significantly decreased protein oxidation by declining the level of circulatory amino acids. However, it did not induce protein synthesis [27].

In a separate study, muscle protein synthesis and mTOR-associated upstream and downstream signalling proteins in young male subjects (*n* = 14) were assessed by using stable isotopic and immunoblotting technique [28]. After the first muscle biopsy, study group ingested a solution enriched with leucine combined with carbohydrate, whereas people in the control group did not consume the nutrients. Following ingestion of solution, a continuous infusion of indocyanine green (ICG) was started in the femoral artery (0.5 mg min^−1^). After 15 minutes of infusion, blood sample was collected from the femoral vein and the arterialized hand vein to measure ICG concentration, blood glucose, insulin, and phenylalanine concentrations. A second biopsy was obtained 1 hour after the infusion of ICG. In the study subjects, leucine rich solution significantly increased muscle protein synthesis, Akt/PKB (protein kinase B), and mTOR phosphorylation and reduced AMPK phosphorylation, whereas in the control group, protein synthesis and cell signalling (phosphorylation status) were not changed (*P* > 0.05). They proposed that anabolic nutrients alter the phosphorylation status of both AMPK- and mTOR-associated signalling proteins in human muscle, in association with an increase in protein synthesis by promoting translation elongation [28].

Sarcopenia is an age related decrease of skeletal muscle mass (SMI) and function [29]. It is a miscellaneous process characterized by alteration in muscle fiber morphology, muscle contractile, and protein kinetics and insulin sensitivity [30]. This incidence occurs due to the derangement between muscle protein synthesis and breakdown [31]. It is discussed above that leucine induces protein synthesis in young adults [28]. In a separate study, twenty-four elderly men (74.3 ± 1.0 y) were randomly assigned to ingest 20 g intrinsically l-[1-^13^C] phenylalanine-labeled casein protein with (supplemented) or without (control) 2.5 g of leucine. The combination of leucine and casein protein enhances the rates of postprandial muscle protein synthesis for up to 6 hours in older men compared to control group [32]. In another study, combined ingestion of leucine (10 g/L) and whey protein (60 g/L) significantly improved the rates of muscle protein synthesis and whole-body protein balance both in older (75 ± 1 years) and young men (20 ± 1 years) [33]. In a clinical study [34], commercially available BCAA supplement (Epic H2O, Williston, VT, USA) was given to female and male subjects who were doing moderate physical activities. This study demonstrates that supplementation of BCAAs with glucose decreases the onset of exercise induced muscle soreness in young females [34].

## 5. BCAAs for Fatigue

Elevated level of brain serotonin is associated with central fatigue [35]. Serotonin cannot cross the blood brain barrier; therefore neurons should synthesize serotonin by themselves. Tryptophan, precursor of serotonin, can cross the blood brain barrier competing with BCAAs since they use the same carrier system [36]. If plasma BCAAs are increased, more BCCAs will be up taken by brain compared to tryptophan and thus improve the condition of fatigue. To investigate whether BCAAs supplementation can improve aerobic performances and ratings of perceived exertion upon exercise, Greer et al. (2011) conducted a study where they have found that BCAAs supplementation was able to reduce perceived exertion; however, it could not attenuate aerobic exercise performance [37].

## 6. BCAAs for Burn, Trauma, and Sepsis

Severe insult like burn, trauma, and sepsis induces marked elevation of protein catabolism and decreases body protein level [38]. Details are given in the following.

### 6.1. BCAAs for Burnt Patients

In an effort to examine the effects of BCAAs on protein synthesis, twenty-two burnt patients were randomly divided into two groups: 11 patients receiving 22% of BCCAs solution and other received 41% of BCAAs solution [39]. Although high BCAAs supplementation markedly decreased both 3MeHis/nitrogen and 3MeHis/creatinine ratio showing an evidence of improving protein catabolism, no improvement was found for nitrogen loss by using the high level BCAAs [39]. In another study, 44% of BCAAs were given to severely burn adult patients. But it was not able to induce protein synthesis on those patients [40].

### 6.2. BCAAs for Trauma

Urea nitrogen excretion is often used as a reliable stress maker. An index of less than zero represents no significant stress, an index of zero to 5 typifies moderate stress, while an index greater than 5 indicates severe stress [41]. Cerra et al. [42] studied the effect of BCAAs on nitrogen retention in surgically stressed patients. Patients having urinary nitrogen excretion of 6–23 g/d were randomized into four groups. Each group of 8 patients received total parenteral nutrition (TPN) containing 15%, 20%, 47%, and 50% of BCAAs. Patients who received higher BCAAs (47% and 50%) had better nitrogen balance at day three. At day seven, patients having 47% BCAAs had better nitrogen balance compared to the other groups [42]. Some other studies also reported that ingestion of BCAAs improves nitrogen balance in surgically stressed patients [43–45].

Since BCAAs catabolism leads to energy metabolism, Jeter et al. [46] investigated the level of plasma BCAAs in human subject within twenty-four hours of mild traumatic brain injury (TBI). They found that BCAAs level was significantly decreased in TBI compared to the healthy individuals. They hypothesized that BCAAs supplementation may decrease TBI pathology by increasing energy content [46]. On a separate trial, Rappaport et al. [47] randomly assigned TBI patients to 15 days of intravenous BCAA supplementation (19.6 g/d) (*n* = 20) or an isonitrogenous placebo (*n* = 20) [48]. Disability rating score (DRS) was measured during admission and 15 days following admission. The DRS is a sensitive, reliable, and valid tool to determine patient's disability following brain injury [47]. They noticed that 15 days after admission, DRS score had improved significantly in both the placebo group (*P* < 0.05 versus baseline) and in the BCAA-supplemented group (*P* < 0.01 versus baseline). The difference between the two groups was also significant (*P* < 0.004). It has been reported that brain injury decreases essential amino acids like tyrosine and tryptophan [49, 50] which are precursor for catecholamines [51]. In the BCAA supplemented group plasma tyrosine concentration improved, whereas tryptophan increased in placebo receiving patients. The authors concluded that BCAAs supplementation improves cognitive functions in TBI patients without having a negative effect on tyrosine and tryptophan [47].

### 6.3. BCAAs for Sepsis

The systemic response to infection caused by microbial invasion is referred to as sepsis [52]. In a prospective, randomized controlled trial, Jimenez Jimenez et al.  (1991) studied the effects of BCAAs on patients with peritonitis [53]. Eighty patients were divided into two groups of 40 patients: one group was getting 45% and the other group received 22.5% BCAA. Patients with 45% of BCAAs had more positive nitrogen balance, significant decrease of stress index, elevation in plasma prealbumin and retinol binding protein levels, an increase in the creatinine/height index, and a more marked fall in the urinary excretion of 3-methylhistidine. However, mortality remained similar between the two supplemented groups [53]. In a separate study, 69 septic patients from seven different university hospitals were randomly divided into three groups based on the TPN administered. Group A (*n* = 22), group B (*n* = 25), and group C (*n* = 22) received 23%, 45%, and 45% of BCAAs, respectively. Although groups B and C received the same amount of BCAAs, their nitrogen intake was different. In both groups B and C, prealbumin and retinol-binding proteins were increased compared to group A. High BCAAs (both groups B and C) level was able to decrease mortality rate compared to group A (*P* < 0.03).

## 7. BCAAs on Diabetes and Cardiovascular Disease

The BCAAs maintain glucose homeostasis by stimulating insulin secretion [54]. However, studies showed that increased circulating level of BCAAs was associated with obesity and insulin sensitivity [55–58]. There is, in fact, a complex relationship between BCAAs and insulin regulation. While Wang et al. (2011) showed that elevation of circulating BCAAs is a significant risk factor for diabetes and insulin resistance [59], another study reported that supplementation of BCAAs actually improves glucoses homeostasis and insulin resistance in hepatic cirrhosis patients [60]. Further investigation is needed to gain better understanding of the role of BCAAs in insulin resistance.

In a recent study, Mels et al. (2013) investigated the association between BCAAs and cardiovascular disease in a biethnic population [61]. 200 African and 209 Caucasian individuals were part of this study. From the blood samples of these individuals, glycated haemoglobin (HbA1c) and BCAAs were assessed. Based on HbA1c value, participants were divided into two groups. The high HbA1c group (>5.6%) had greater Ambulatory blood pressure (BP), carotid intima-media thickness (cIMT), and BCAAs (all *P* < 0.001). In both groups, there was a significant positive association between ambulatory blood pressure and cIMT with BCAAs (all *P* < 0.05). Thus, the authors demonstrated that BCAAs are independently related to ambulatory BP and cIMT in individuals with high HbA1c levels [61].

In another study, Bhattacharya et al. (2013) investigated the association of BCAAs and other metabolites with coronary artery disease (CAD) [62]. They have conducted mass-spectrometry-based profiling of 63 metabolites in fasting plasma from 1983 sequential patients who were undergoing cardiac catheterization. Study subjects were divided into two groups based on their CAD index which is a numeric summary of angiographic extent of CAD. Patients having CAD index >32 (at least one epicardial coronary artery with 95% stenosis) were termed as case, whereas CAD index < 23 (no individual epicardial coronary artery with >50% stenosis) were termed as the controls. Upon covariate adjustment, the severity of CAD, which is measured by number of diseased vessels, was found to be associated with factor 7 (short-chain acylcarnitines, *P* = 0.0003) and factor 10 (BCAA, *P* = 0.01). By this study, the authors have validated that BCAAs and its related metabolites are independently associated with CAD severity [62].

## 8. BCAAs in Maple Syrup Urine Disease

Maple syrup urine disease (MSUD) is an inherited disorder characterized by accumulation of BCAAs in urine having odor of maple syrup. Individuals, who suffer from MSUD, have mutation on branched-chain *α*-keto acid dehydrogenase (BCKDH) complex. BCKADH (EC 1.2.4.4) is a multienzyme complex located on the inner surface of the inner mitochondrial membrane that catalyzes the oxidative decarboxylation of a-ketoisocaproate, a-keto-methylvalerate, and a-ketoisovalerate and is also involved in the metabolism of BCAAs [1]. In vitro study by Ribeiro et al. revealed that BCAAs reduce the activity of mitochondrial complex leaving more reactive oxygen species in the tissue [63]. This indicates that the major metabolites accumulating in MSUD disturb brain aerobic metabolism by compromising the citric acid cycle and the electron flow through the respiratory chain. For the management of MSUD, leucine is often restricted, but for net protein synthesis in tissues insulin, free amino acids, isoleucine, and valine are supplemented [64].

## 9. Administration of BCAAs in Renal Disease

Elderly patients on chronic haemodialysis are frequently suffering from malnutrition [65]. Irrespective of haemodialysis, patients with renal failure have low level of circulatory essential and nonessential amino acids [66, 67]. Since there is a positive association between plasma BCAAs and appetite [68], Hiroshige et al. investigated the effects of oral administration of BCAAs on overall nutritional status on patients undergoing chronic haemodialysis [69]. In their study, 28 malnourished patients were assigned to receive either placebo or BCAAs supplementation for six months; after that treatment was reversed between the groups. Supplemented group was getting 4 g of BCCAs with 2 g of dextrose at a time, whereas placebo group received 6 g of dextrose. After six months of BCCAs supplementation anorexia reduced and overall nutritional status improved whereas placebo did not have any effect on nutritional status [69].

## 10. BCAAs on Mitochondrial Biogenesis

There is a strong relationship between mitochondrial biogenesis and eukaryotes survival rate. It has been reported that BCAAs oral supplementation (1.5 mg/g body weight/day beginning at 9 months) stimulated mitochondrial biogenesis and increased the average life span of middle aged mice, whereas the same supplementation did not exert any effect on young mice aged 4–6 months [70]. At the molecular level, the BCAA supplementations have shown to be associated with the activation of mTOR in HL-1 cardiomyocytes. The mTOR is a major regulator of protein synthesis in response to exogenous supplementation of amino acids which, in turn, increases the mitochondrial gene expression through the action of PGC-1*α* and partly through increasing the NO generating system and decreases in the production of reactive oxygen species (ROS) by upregulating the ROS defense system in adult male mice. This makes BCAA supplementation a promising candidate to promote the health condition of aged human patients. The role of BCAAs supplementation in mitochondrial biogenesis has been extensively discussed elsewhere [71].

## 11. Conclusion 

Branched-chain amino acids have been used as dietary supplements for various pathophysiological conditions. Even though their role in protein synthesis is not consistent across different studies, it is clear that BCAAs can reduce the negative nitrogen balance in the living system. Evidences have shown that BCAAs can prevent muscle loss in both young and elderly people. In addition, BCAA-supplementation has benefit to patients with liver cirrhosis and liver cancer. BCAAs have also been used on critically ill patients with severe burn, sepsis, surgery, and trauma in different studies. Some of those studies have found beneficial effect after BCAA administration while some did not. However, BCAAs administration did not worsen the condition in any of the cases. Our current knowledge regarding the role of BCAA in insulin regulation is still not clear. It has been reported that increased BCAAs level is associated with CVD, diabetes, and MSUD. But if an individual gets a diet which is low in BCAAs it can affect the overall body protein synthesis, insulin regulation, glucose homeostasis, glutamate-glutamine pool, and antioxidant level. Therefore, further studies are needed to fill up the knowledge gap between BCAAs metabolism and the related regulation of these amino acids.
